# The effect of intracerebroventricular administration of orexin receptor type 2 antagonist on pentylenetetrazol-induced kindled seizures and anxiety in rats

**DOI:** 10.1186/s12868-018-0445-9

**Published:** 2018-08-13

**Authors:** Saeedeh Asadi, Ali Roohbakhsh, Ali Shamsizadeh, Masoud Fereidoni, Elham Kordijaz, Ali Moghimi

**Affiliations:** 1 Department of Biology, Rayan Center for Neuroscience and Behavior, Faculty of Science, Ferdowsi University of Mashhad, P.O. Box 9177948974, Mashhad, Iran; 2 Pharmaceutical Research Center, Pharmaceutical Technology Institute, Mashhad University of Medical Sciences, Mashhad, Iran; 3 Physiology-Pharmacology Research Center, Rafsanjan University of Medical Sciences, Rafsanjan, Iran

**Keywords:** Anxiety, Kindling, Orexin, Orx2 receptor, PTZ, Seizure

## Abstract

**Background:**

Current antiepileptic drugs are not able to prevent recurrent seizures in all patients. Orexins are excitatory hypothalamic neuropeptides that their receptors (Orx1R and Orx2R) are found almost in all major regions of the brain. Pentylenetetrazol (PTZ)-induced kindling is a known experimental model for epileptic seizures. The purpose of this study was to evaluate the effect of Orx2 receptor antagonist (TCS OX2 29) on seizures and anxiety of PTZ-kindled rats.

**Results:**

Our results revealed that similar to valproate, administration of 7 µg/rat of TCS OX2 29 increased the latency period and decreased the duration time of 3rd and 4th stages of epileptiform seizures. Besides, it significantly decreased mean of seizure scores. However, TCS OX2 29 did not modulate anxiety induced by repeated PTZ administration.

**Conclusion:**

This study showed that blockade of Orx2 receptor reduced seizure-related behaviors without any significant effect on PTZ-induced anxiety.

## Background

Epilepsy is a chronic neurological disorder that its main characteristic is the recurrent appearance of spontaneous seizures [1]. Research on experimental models of this disease indicates that there is an imbalance between the inhibitory GABAergic and excitatory glutamatergic neurotransmission in the central nervous system (CNS) [2]. At present, various antiepileptics are available. However, dose-related neurotoxicity, a range of drug interactions, and systemic side effects are the major problems caused by current antiepileptic drugs [3].

Orexins (orexin A and B) are hypothalamic excitatory neuropeptides [4]. Orexinergic neurons are located in the lateral hypothalamic area, perifornical area, dorsomedial hypothalamus, and posterior hypothalamus, which project to different parts of the brain [5]. The physiological functions of the orexins are mediated by two G-protein coupled receptors: orexin receptor type 1 (Orx1R) and orexin receptor type 2 (Orx2R). The affinity of Orx1R for orexin A is higher than orexin B, whereas Orx2R has a similar affinity for both neuropeptides [6]. Stimulation of these receptors increases intracellular Ca^2+^ through Gq/11 activation in orexin responsive cells [7]. It was demonstrated that the activation of orexin receptors provoked cortical pyramidal cells and enhanced cortical excitability [8, 9]. Orexin A was reported to be involved in long-term potentiation of synaptic transmission in the CA1 region of the hippocampus. This effect was dependent on ionotropic and metabotropic GABAergic, glutamatergic, as well as cholinergic and noradrenergic receptors implying the active role of the orexinergic system in learning and memory [10]. Similarly, Riahi et al. [11] showed that administration of orexin A in the lateral ventricle of the rats increased electrical activity of the hippocampal pyramidal neurons.

Orexins regulate the release of serotonin, gamma-aminobutyric acid (GABA), and glutamate [12, 13]. These neurotransmitters are involved in the regulation of sleep and wakefulness, pain, food intake, reward, multiple sclerosis, and stress [5, 14]. As mentioned, orexins have excitatory effects in the CNS. There is evidence implying that orexins may be involved in the generation and propagation of seizures. For example, it was reported that intracortical and intracerebroventricular injections of orexins caused seizure-related behaviors in rats [15, 16]. Both orexin A and B enhanced the excitability of the central nervous system following administration of penicillin G [17]. In accordance, it was revealed that during pilocarpine-induced epileptic activity, the expression of orexin B was increased in the rat hippocampus [18].

Chemical kindled seizure is an animal model of temporal lobe epilepsy induced by repeated administration of an initially subconvulsive chemical stimulus such as pentylenetetrazol (PTZ) that results in behavioral signs of tonic and clonic seizures [19]. Injection of such chemicals decreases seizure threshold and culminates in a generalized seizure [20]. In other words, PTZ increases seizure susceptibility. The molecular mechanism(s) behind this phenomenon has not been well understood. Some studies have offered that inhibition of main inhibitory systems of the CNS including GABAA-mediated actions and activation of stimulatory systems such as NMDA, AMPA, and kainate receptors are part of a complex network that culminates in the development of kindling [21, 22]. Kindling has been introduced as a reliable experimental model for complex partial epilepsy in patients [23] and has been considered as a drug-resistant model of epilepsy [24].

On the other hand, anxiety is a common comorbidity that is related to epilepsy [25]. It has been demonstrated that the orexinergic system and the hypothalamic–pituitary–adrenal axis contribute together in the modulation of stress responses [26]. Moreover, orexins exert anxiety both in mice and rats [27, 28]. Using optogenetic approaches, Sears et al. [29] showed that stimulation of orexin fibers in the locus coeruleus increased threat memory formation induced via an auditory stimulus. Also, it was demonstrated that orexin A levels in the amygdala were increased following social interaction, positive emotions, and anger in narcoleptic patients [30]. Another study showed that there was a positive relation between orexin levels and childhood maltreatment [31]. All these studies imply that orexinergic system has an important role in the modulation of anxiety both in rodents and human. On the other hand, orexin is an important neuropeptide with significant effects on food intake. It increases appetite and consequently food intake. As nutrition has been reported to be involved in anxiety [32], it is a possibility that orexins and their antagonits, through alteration of appetite, modulate anxiety.

On the basis of these findings, we hypothesized that blockade of Orx2 receptors might be useful for the prevention of epilepsy and concomitant anxiety. Accordingly, we aimed to assess the effect of an Orx2R antagonist (TCS OX2 29) on PTZ-induced chemical kindling and anxiety.

## Methods

### Drugs

Orexin antagonist (TCS OX2 29) was purchased from Tocris (Bristol, UK). Its chemical name is (2S)-1-(3,4-Dihydro-6,7-dimethoxy-2(1H)-isoquinolinyl)-3,3-dimethyl-2-[(4-pyridinylmethyl)amino]-1-butanone hydrochloride. This drug was first introduced by Hirose et al. in 2003. After that, the drug has been used widely as a selective Orx2R antagonist. It promoted sleep [33], decreased heart rate, and blood pressure [34], reduced morphine place preference [35], prevented analgesia, and reduced alcohol self administration [36].

Pentylenetetrazol and sodium valproate were purchased from Sigma (India) and Sanofi-aventis (France), respectively. PTZ and sodium valproate were dissolved in 0.9% sterile saline. TCS OX2 29 was dissolved in dimethyl sulfoxide (DMSO), tween 80 and sterile 0.9% saline (10/10/80% v/v respectively).

### Animals

Adult male Wistar rats (200–250 g) were used in this study. The animals were bred in the experimental animal house of Ferdowsi University. All animals were maintained under normal conditions (12/12 h light/dark cycle, temperature: 23 ± 2 °C), with ad libitum availability of food and water. Each experimental group included seven animals.

### Stereotaxic surgery and microinjections

Rats were anesthetized with an intraperitoneal (IP) administration of ketamine (100 mg/kg) and xylazine (4 mg/kg). They were fixed on a stereotaxic apparatus (Narishige, Tokyo, Japan). Enrofloxacin was injected to prevent infections and ketoprofen was administrated for post-operative analgesia. Stainless steel guide cannula (22-gauge) fitted with the infusion cannula (27-gauge, 1 mm longer) was implanted into the left lateral ventricle (AP = 0.8 mm, ML = 1.6 mm and D = 4 mm) according to Paxinos and Watson’s stereotaxic atlas [37]. Acrylic dental cement and surgical screws were used to fix the guide cannula. Six days after recovery from the stereotaxic surgery, different solutions (TCS OX2 29, valproate and vehicle) were injected into the left lateral ventricle (2 μl/rat) of awake and freely moving rats. Intracerebroventricular (ICV) injections were done using an infusion pump (Stoelting, USA) at the rate of 2 μl/min. At the end of experiments, 0.5 μl of methylene blue was injected through the guide cannula. After that, the rats were killed and the brain slices prepared using microtome checked under a stereo microscope to ensure the placement of cannula.

### Induction of kindling and experimental design

For induction of kindling, PTZ (32 mg/kg) was injected intraperitoneally every other day for 23 days [38].

The vehicle (2 μl/rat), TCS OX2 29 (1, 3.5 and 7 μg/rat) and valproate (as the control drug, 26 μg/rat), were administered ICV 30 min before PTZ injections. The doses for TCS OX2 29 and valproate were selected according to previous studies [4, 39]. After injection of PTZ, seizure-related behaviors were recorded for 30 min. The intensity of seizure behaviors was recorded on the following scale: 0 = no response; 1 = vibrissae twitching, mouth and facial jerks; 2 = myoclonic body jerks or head nodding; 3 = forelimb clonus; 4 = rearing, falling down, forelimb tonus and hindlimb clonus; and 5 = tonic extension of the hindlimb, status epilepticus [40, 41]. Accordingly, we recorded the following parameters: (1) Median of seizure scores (2) Latency to the first forelimb clonus (S3L) (3) Duration of forelimb clonuses (4) Latency to the first sign of scale 4 behaviors (S4L) (5) Duration of scale 4 behaviors (S4D).

### Elevated plus-maze (EPM)

EPM test is a standard method that has been employed for determining the anxious behaviors in rodents [42]. Hereafter, anxious behaviors in rats referred to as anxiety. It consists of two open and two closed arms as a plus sign. The percentage of open arm entries (%OAE) and the percentage of time spent on the open arms (%OAT), as standard indices of anxiety, were recorded for 5 min. Total arm entries were also recorded as a measure of spontaneous locomotor activity. A significant increase in %OAT and %OAE represent a lower anxiety response [43].

On the last day of the experiments (30 min after PTZ injection), animals were placed on the EPM. To compare the effect of PTZ on anxiety, an extra control group of animals received TCS OX2 29 vehicle (2 µl/rat, ICV) and saline (6 ml/rat, IP) and were tested in the EPM.

### Statistical analysis

The data for seizure stages were expressed as median ± interquartiles. The stages were analyzed using Kruskal–Wallis non-parametric one-way analysis of variance (ANOVA) followed by 2-tailed Mann–Whitney’s U test. Other data were expressed as mean ± SEM. Differences between groups were analyzed by one-way analysis of variance which was followed by Tukey as post-test. The minimum level of significance was set at P < 0.05.

## Results

### The effects of TCS OX2 29 on chemical kindling

**Fig. 1:**
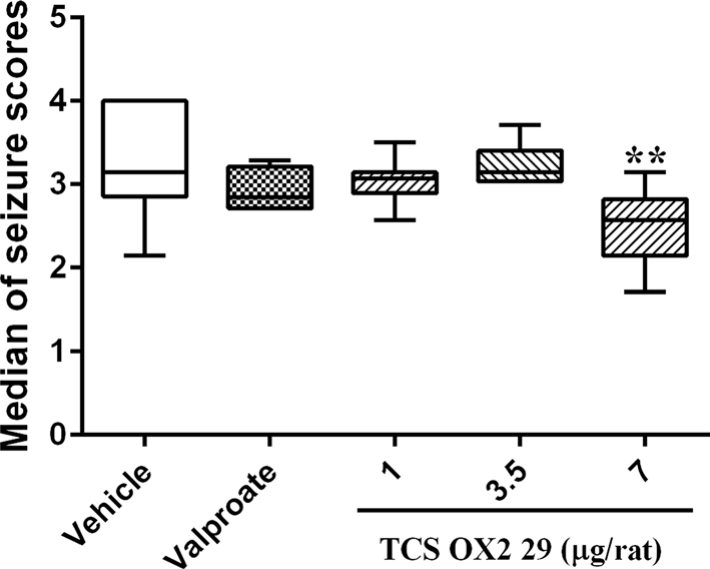
The effect of intracerebroventricular injection of TCS OX2 29 (1, 3.5 and 7 μg/rat) and valproate (26 µg/rat) on median of seizure scores in pentylenetetrazol-kindled rats. TCS OX2 29 at the dose of 7 μg/rat reduced median of seizure scores. Each bar represents median ± interquartiles. **P < 0.01 compared to vehicle-treated rats. In each group n = 7

The results showed that administration of TCS OX2 29 at the dose of 7 μg/rat significantly decreased median of seizure scores (P < 0.01). However, TCS OX2 29 at the doses of 1 and 3.5 μg/rat and valproate did not significantly change the median of seizure scores (Fig. 1).

**Fig. 2:**
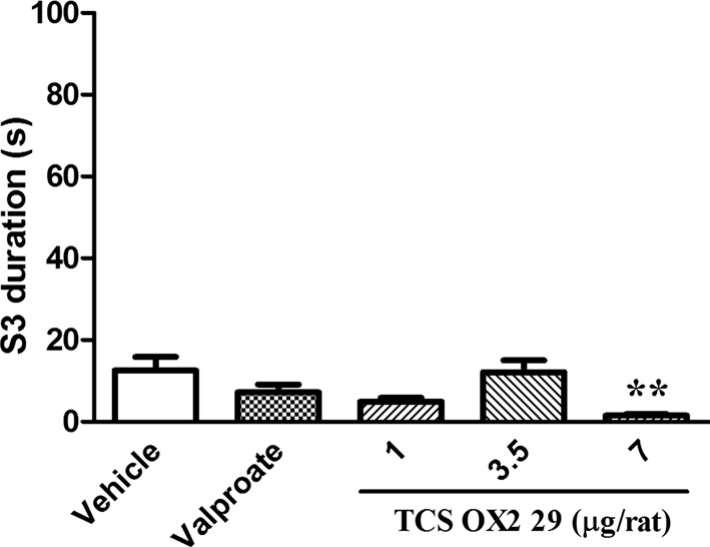
The effect of intracerebroventricular injection of TCS OX2 29 (1, 3.5 and 7 μg/rat) and valproate (26 µg/rat) on stage 3 duration in pentylenetetrazol-kindled rats. TCS OX2 29 at the dose of 7 μg/rat reduced the stage 3 duration. Each bar represents mean ± SEM. **P < 0.01 compared to vehicle-treated rats. In each group n = 7

**Fig. 3:**
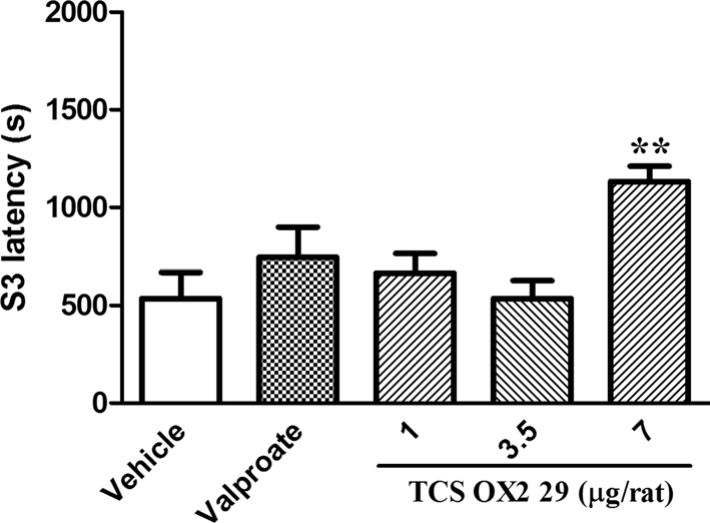
The effect of intracerebroventricular injection of TCS OX2 29 (1, 3.5 and 7 μg/rat) and valproate (26 µg/rat) on stage 3 latency in pentylenetetrazol-kindled rats. TCS OX2 29 at the dose of 7 μg/rat increased latency of 3th stage of seizures. Each bar represents mean ± SEM. **P < 0.01 compared to vehicle-treated rats. In each group n = 7

Furthermore, TCS OX2 29 at the dose of 7 μg/rat decreased the duration of the 3rd stage of seizure (S3 duration, P < 0.01) and increased the stage 3 latency (S3 latency, P < 0.01), in comparison with the control group. However, valproate failed to show such protective effects either on S3 duration or S3 latency (Figs. 2, 3).

**Fig. 4:**
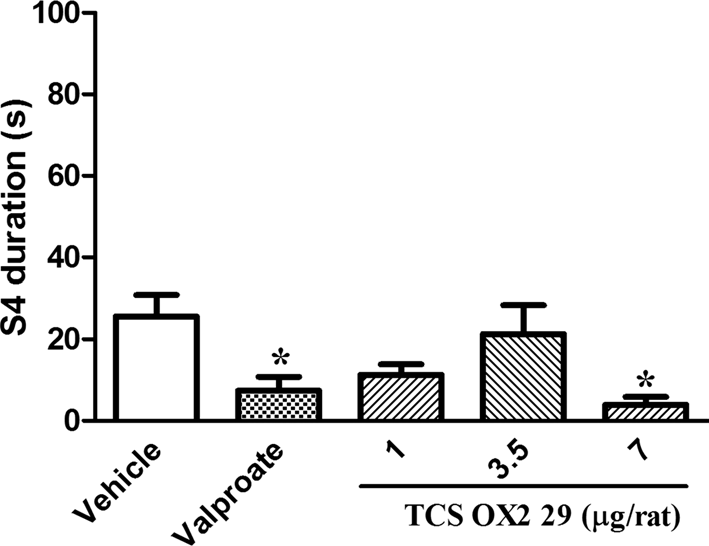
The effect of intracerebroventricular injection of TCS OX2 29 (1, 3.5 and 7 μg/rat) and valproate (26 µg/rat) on stage 4 duration in pentylenetetrazol-kindled rats. TCS OX2 29 at the dose of 7 μg/rat and valproate at the dose of 26 μg/rat decreased duration of 4th stage of seizures. Each bar represents mean ± SEM. *P < 0.05 compared to vehicle-treated rats. In each group n = 7

**Fig. 5:**
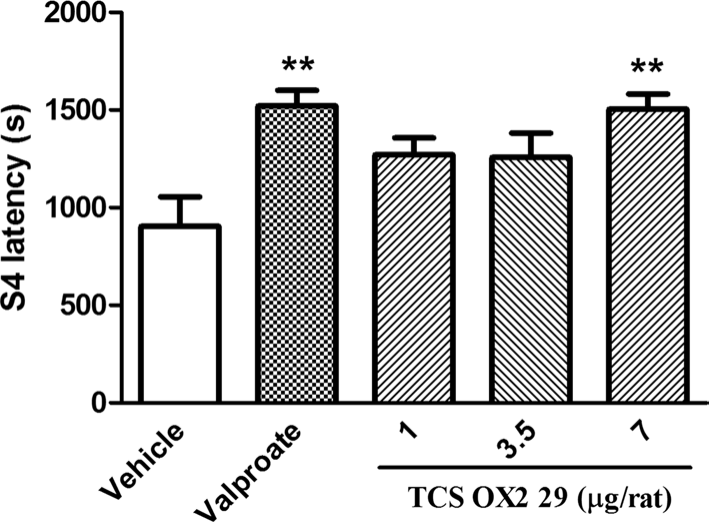
The effect of intracerebroventricular injection of TCS OX2 29 (1, 3.5 and 7 μg/rat) and valproate (26 µg/rat) on stage 4 latency in pentylenetetrazol-kindled rats. TCS OX2 29 at the dose of 7 μg/rat and valproate at the dose of 26 μg/rat increased the latency period of stage 4 seizures. Each bar represents mean ± SEM. **P < 0.01 compared to vehicle-treated rats. In each group n = 7

TCS OX2 29 at dose of 7 μg/rat also decreased the duration of the 4th stage of seizure (S4 duration, P < 0.05) and increased the stage 4 latency (S4 latency, P < 0.01). Similarly, valproate decreased S4 duration (P < 0.05) and increased S4 latency (P < 0.01). TCS OX2 29 at doses of 1 and 3.5 μg/rat had no significant effect on either S4 duration or S4 latency (Figs. 4, 5).

### The effects of TCS OX2 29 on PTZ-induced anxiety

**Fig. 6:**
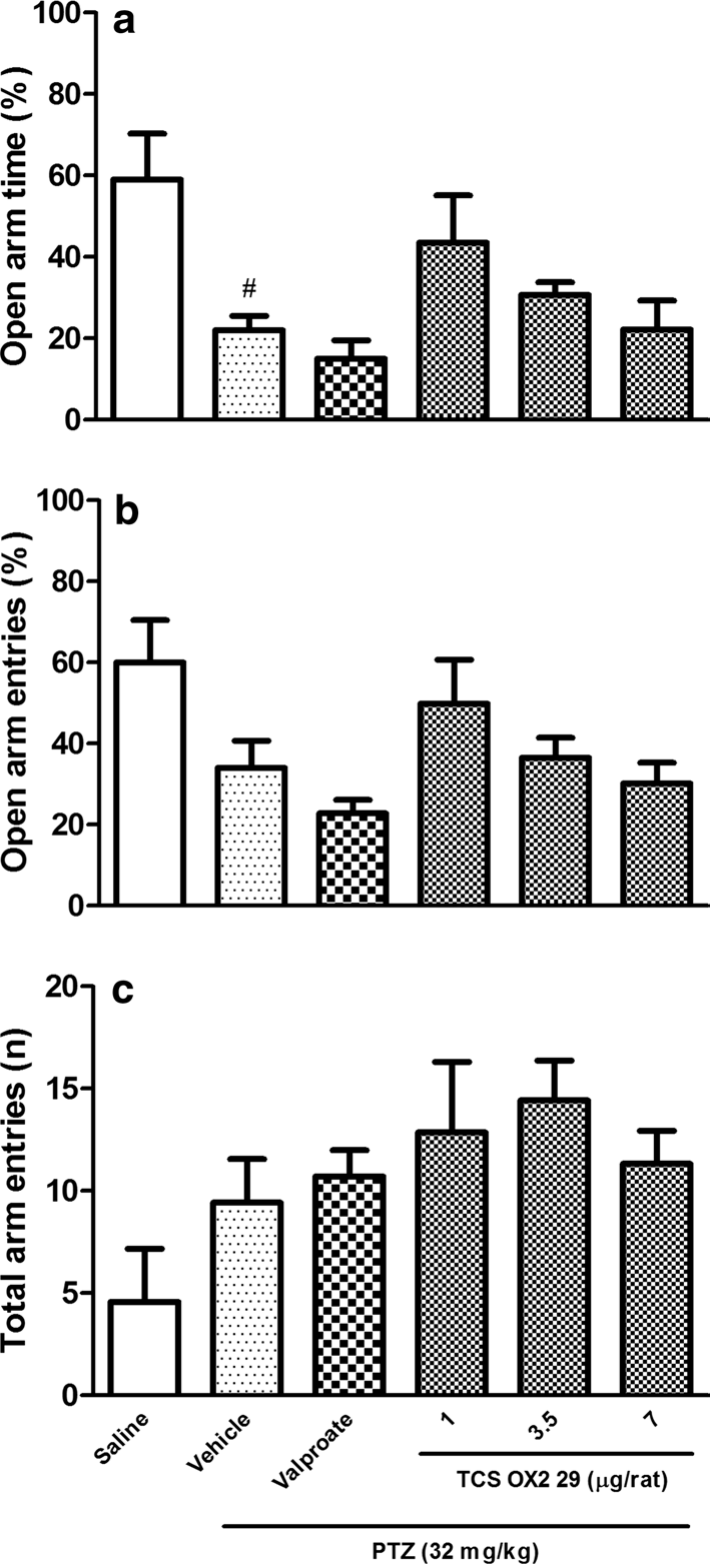
**a**, **b** The effect of intracerebroventricular injection of TCS OX2 29 (1, 3.5 and 7 μg/rat) and valproate (26 µg/rat) on anxiety of PTZ-kindled rats. This figure shows PTZ induced anxiety that was not reversed either by TCS OX2 29 (1, 3.5 and 7 μg/rat) or valproate (26 μg/rat). **c** Shows that the locomotor activity of all groups were similar. Each bar represents mean ± SEM. #P < 0.05 compared to the saline-treated rats. n = 7. *PTZ* pentylenetetrazol

The results of EPM test showed that kindling induced anxiety that was manifested as the diminished percentage of time spent on the open arms (P < 0.05) in vehicle/PTZ-treated rats. In these animals, the locomotor activity was not different from the control group implying that PTZ induced an anxiety. However, administration of either valproate or TCS OX2 29 (1, 3.5 and 7 μg/rat) did not change the anxiety of PTZ-kindled rats (Fig. 6a–c).

## Discussion

The results of this study showed that similar to valproate, intracerebroventricular administration of TCS OX2 29 at the highest dose (7 μg/rat) induced significant anti-seizure effects on generalized convulsions in PTZ-kindled rats. For the first time, the results of our study revealed that Orx2R antagonists have the potential to be used in the prevention of partial seizures with secondary generalization. However, Orx2R antagonist failed to resolve concomitant anxiety.

It is presumed that blockade of the GABAergic system and increased activity of the glutamatergic system are neuronal processes involved in the kindling [19, 44]. Previous studies revealed that orexin A and orexin B have stimulatory effects on the neuronal system. For example, Stanley and Fadel showed that injection of orexin A into the CA1 area of the hippocampus increased glutamate release [45]. Similarly, it was demonstrated that following orexins injection into the cerebrospinal fluid, glutamate level in the hippocampus was increased, and it was reduced after administration of Orx1R antagonist. Similar to these findings, Goudarzi et al. [46] showed that TCS OX2 29 reduced convulsive stages and duration. There is more evidence to support our results; in a recent study, it was demonstrated that administration of TCS OX2 29 reduced the severity of seizures and neuronal damage in the hippocampus of the rats following PTZ administration and sleep deprivation [4]. Also, the orexinergic system influenced the function of the limbic structures and the neocortex which are involved in controlling the incidence of seizures and epilepsy through their projections to the neuromodulatory centers located in the brain stem [47, 48]. The high-density expression of Orx2R in CA3 area of the hippocampus [49], can be a reason for the pro-convulsant effect of the orexins [50]. Furthermore, according to the previous evidence, orexin may also induce its stimulatory effects through the modulation of the GABAergic system. It is known that orexin affects the release of GABA [51] and upregulates mechanisms responsible for the synthesis and the release of glutamate [52]. In accordance, Goudarzi et al. [46] showed that following hippocampal Orx1R but not Orx2R blockade, the release of GABA was increased. Similarly, a dual orexin receptor antagonist increased the activity of GABAergic systems in the basal forebrain [51]. On the other hand, glutamate and GABA increase and decrease the activity of orexinergic neurons, respectively [53].

Another possibility that explains the effects of TCS OX2 29 on seizure is the activation of two distinct pathways by Orx2R. It was revealed that orexinergic receptors are present on different cortical GABAergic interneurons [54, 55] and neocortical pyramidal cells [56]. Here, we suggest that there are connections between some cortical inhibitory interneurons and cortical pyramidal neurons (e.g., hippocampus). In our proposed mechanism, in spite of inhibitory actions of interneurons on pyramidal neurons, during activation of orexin receptors present on both neurons, pyramidal neurons will have normal outputs. In this study, TCS OX2 29 at the dose of 1 μg/rat reduced the pyramidal neurons output after which the seizure threshold increased. However, TCS OX2 29 at the dose of 3.5 μg/rat inhibited pyramidal neurons but exerted more inhibitory effects on the interneurons, so the output of pyramidal neurons increased followed by increased susceptibility to convulsions. This suggestion may be confirmed by Tang et al. [57] study that showed the concentration dependent inhibition of Gq or Gi proteins by Orx2R antagonists, which induced different effects. Hence, at the dose of 3.5 μg/rat of the Orx2R antagonist, stronger inhibition of interneurons may be explained by more coupling of orexin receptors with Gi rather than Gq. At the dose of 7 μg/rat, the antagonist may increase the tendency of interneurons receptors for coupling with Gq (instead of Gi) that will diminish pyramidal neurons outputs and convulsive behaviors. It is possible that orexins, by activation of either Orx1R or Orx2R, modulate the function of neurotransmitters other than GABA and glutamate. Such interaction was reported just recently for endocannabinoids as TCS OX2 29 enhanced the effect of a cannabinoid receptor antagonist in the conditioned place preference paradigm [58].

Although we choose the dose of valproate as a standard antiepileptic drug according to a previous report [39], it did not induce a robust anti-seizure effect in the 3rd stage of seizure in the this study. In clinical practice, valproate has been used extensively for the treatment of various kinds of epilepsy including partial seizures. Valproate, via diverse pharmacological actions including the modulation of Na^+^ channels, inhibition of Ca^2+^ channels, inhibition of GABA transaminase, and increase in GABA concentration induces anticonvulsant effects [59]. However, there are reports showing that valproate has lower efficacy than carbamazepine, as an standard antiepileptic drug, in the treatment of partial and secondary generalized tonic–clonic seizures. This may explain the low efficacy of valproate in this study [60, 61]. Stanojlović and colleagues showed that valproate rapidly reduced mean seizure score and audiogenic convulsions in metaphit-treated Wistar rats. However, the drug did not exhibit significant effect on electrocortical activity. So, they concluded that valproate is an anticonvulsant rather than antiepileptic drug [62]. By comparison, it may be suggested that TCS OX2 29 had a higher potency than valproate.

Epileptic patients also suffer from anxiety [25]. So, finding medications that are able to treat both disorders are of great interest and importance. Our obtained results revealed that PTZ induced anxiety in the elevated plus-maze. In parallel with our results, it was demonstrated that PTZ, by activation of the glutamatergic system, induced convulsion and caused anxiety [63]. We presumed that orexinergic system is a target that modulates both anxiety and seizure. It has been reported that orexinergic system overactivation is an important factor in maintaining arousal and anxiety [64]. It was reported that depressive behaviors were higher in mice with lower hippocampal orexin [65]. In addition, knocking down of Orx2R in the basolateral amygdala increased anxious behaviors in mice [66]. Approval of suvorexant, as a dual receptor antagonist, for the treatment of insomnia [67] shows the importance of the orexinergic system in sleep and wake cycle. There are numerous orexinergic terminals in areas associated with stress and anxiety including the middle prefrontal cortex of cingulate cortex. Also, ICV injection of orexin A induced anxiety in different experimental models of anxiety [28]. Similarly, it was reported that injection of orexin A and B in the paraventricular nuclei of the thalamus caused anxiety [27]. This evidence implies that orexinergic system is an important target in the modulation of stress and anxiety [68]. However, it should be mentioned that previous studies showed that Orx1R has a more important role than Orx2R in the modulation of anxiety [64]. According to our results, TCS OX2 29 failed to overcome PTZ-induced anxiety. This finding is possibly in accordance with previous studies showing that the anxiogenic effect of orexins is mediated mainly by Orx1R [69]. However, we cannot rule out the effect of TCS OX2 29 on anxiety at higher doses. Our results also revealed that the motor activity of the animals that received TCS OX2 29 was not different from the vehicle-treated group on the EPM. This finding may imply that the anticonvulsant effect of TCS OX2 29 at the dose of 7 µg/rat was not influenced by changes in muscle tone. One of the limitations of this study is that we used an animal model of anxiety that did not have a social component. For example, social interaction test would be more applicable in such kind of studies. As the second limitation, electrical kindling has been reported with minor advantages over chemical kindling that makes it a better method for evaluation of the potential anticonvulsant drugs [70]. Finally, it is a possibility that orexin and its antagonists modulate epilepsy and anxiety via metabolic changes. For example, hyperhomocysteinemia is reported to be affected by different dietary patterns [71]. Hyperhomocysteinemia has been related to anxiety-like behaviors in rats [72] and human [73]. On the other hand, low levels of homocysteine may induce epilepsy [74]. Considering the very important role of orexinergic system in feeding and nutrition, it is a possibility that this system modulates anxiety and/or epilepsy via metabolic changes.

Our study showed that TCS OX2 29, as a selective Orx2R antagonist, reduced the severity of the seizures of the PTZ-kindled rats. However, it did not affect PTZ-induced anxiety.

## Conclusion

It may be suggested that the orexinergic system has the potential to be considered as an important target in the treatment of epilepsy.
